# Gut commensals require Peyer’s patches to induce protective systemic IgA responses

**DOI:** 10.21203/rs.3.rs-4220532/v1

**Published:** 2024-05-13

**Authors:** Joshua R. Harris, Victoria Zoccoli-Rodriguez, Mara S. Delaney, Tania N. Cruz, Brian T. Gaudette, Joel R. Wilmore

**Affiliations:** 1 Department of Microbiology and Immunology, SUNY Upstate Medical University, Syracuse, NY,; 2 Department of Inflammation and Immunity, Lerner Research Institute, Cleveland Clinic, Cleveland, OH.; 3 Sepsis Interdisciplinary Research Center, SUNY Upstate Medical University, Syracuse, NY.

## Abstract

Gut educated IgA secreting plasma cells that disseminate beyond the mucosa and into systemic tissues have been described as providing beneficial effects from disease in several contexts. Several bacteria have been implicated in the induction of systemic IgA, however the mechanisms that result in differential levels of induction by each bacterial species are still unknown. Here we show, the commensal bacteria, *Bacteroides fragilis* (*Bf*), is an efficient inducer of systemic IgA responses. The ability of *Bf* to induce the production of bone marrow IgA plasma cells and high levels of serum IgA relied on high levels of gut colonization in a dose-dependent manner. Colonization induced *Bf*-specific IgA responses were severely diminished in the absence of Peyer’s patches, but not the murine cecal patch. Colonization of mice with *Bf*, a natural human commensal, resulted in few changes within the microbiome and the host transcriptional profile in the gut, suggesting a commensal relationship with the host. *Bf* colonization did benefit the mice by inducing systemic IgA that led to increased protection in a bowel perforation model resulting in lower peritoneal abscess formation. These findings demonstrate a critical role for bacterial colonization and Peyer’s patches in the induction of robust systemic IgA responses that confer protection from bacterial dissemination outside of the gut.

## Introduction

Proper immunological development is dependent on the presence of gastrointestinal microbes that have co-evolved with their hosts to establish symbiotic homeostasis^1^. A primary mediator of this relationship is mucosal (secretory) IgA, which generally functions to exclude pathogens and foster colonization by beneficial microbes^2^. Although decades of research have uncovered myriad roles for secretory IgA at the mucosa, less is known about the mechanisms that lead to the induction and generation of systemic IgA and its significance to overall health. The generation of IgA-secreting plasma cells that disseminate to systemic sites is directly dependent on which microbial species colonize the gastrointestinal tract^3–5^. However, the precise mechanisms that allow certain species to induce systemic IgA responses are largely unknown. Importantly, emerging studies have begun to illustrate the multifaceted importance of systemic IgA plasma cells and serum IgA, including in protection against pathologies such as polymicrobial sepsis and meningoencephalitis^3,6^.

Paradoxically, microbes that demonstrate high immunogenic capacity and symbiotic characteristics, can also display pathogenic potential when mucosal-barrier integrity is compromised^7–9^. One such example is *Bacteroides fragilis* (*Bf*), a ubiquitous Gram-negative anaerobe with a highly immunogenic capsular polysaccharide coat (CPC^)10.^ The polysaccharide A (PSA) component of the *Bf* CPC has been extensively shown to increase anti-inflammatory IL 10 secretion that is protective in models of colitis and autoimmune encephalitis^11–12^. *Bf* induces robust secretory IgA responses that facilitate intimate mucosal colonization and support exclusion of pathogenic invading microbes^13^. Despite these symbiotic roles, *Bf* is implicated in numerous pathologies, particularly bacteremia, sepsis, and peritoneal abscess formation following escape from the intestinal compartment^14^. Here we show that gut colonization by *Bf* leads to robust systemic IgA responses that are protective against abscess formation. This protective systemic IgA response is T cell-dependent and requires the presence of Peyer’s patches. This study lends to the emerging pattern in which some microbes that colonize close to their host intestinal epithelial layer demonstrate a high propensity to elicit homeostatic mucosal and systemic IgA^15–16^. Our work uncovers a dynamic in which hosts may have evolved microbe-specific systemic IgA responses as a fail-safe mechanism to ensure additional protection from closely associated mucosal microbes to protect against circumstances in which barrier integrity is lost.

## Results

### *Bacteroides fragilis* induces systemic IgA

**Extended Data Fig. 1.**
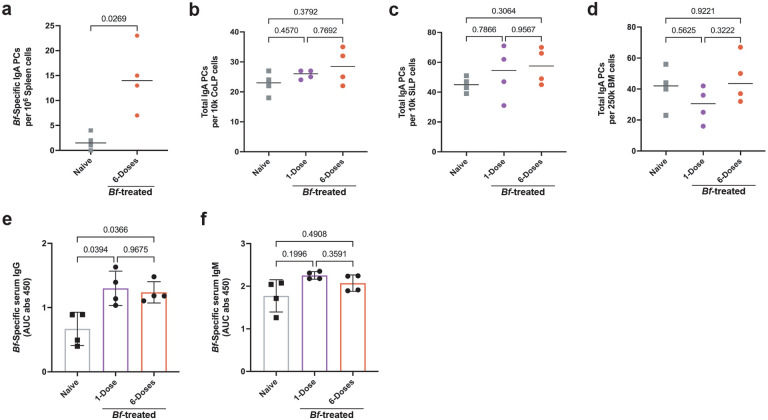
*Bf*-Systemic IgA induction does not alter total IgA levels. **a**, Detection of *Bf*-specific IgA plasma cells in the spleen in 6-dose treated mice as determined by ELISpot using plates pre-coated in *Bf*-antigen. **b**,**c**,**d**, ELISpot assays to determine total IgA plasma cell populations in colonic (**b**) and small intestinal (**c**) lamina propria and bone marrow (**d**) using ELISpot plates pre-coated with anti-Ig(H&L) and developed using anti-IgA-specific antibodies. **e**,**f**, Serum ELISA determined the level of *Bf*-specific IgG (**e**) and IgM (**f**) in *Bf*-treated mice 6-weeks after initial dose. For serum ELISA, the area under the curve (AUC) was calculated based on the absorbance at 450 nm from each serially diluted sample. Statistical analysis was performed using students t-test with Welch’s correction (**a**) or one-way analysis of variance (ANOVA) with Turkey multiple-comparison test (**b**-**f**). Exact *P* values are shown.

**Fig. 1.**
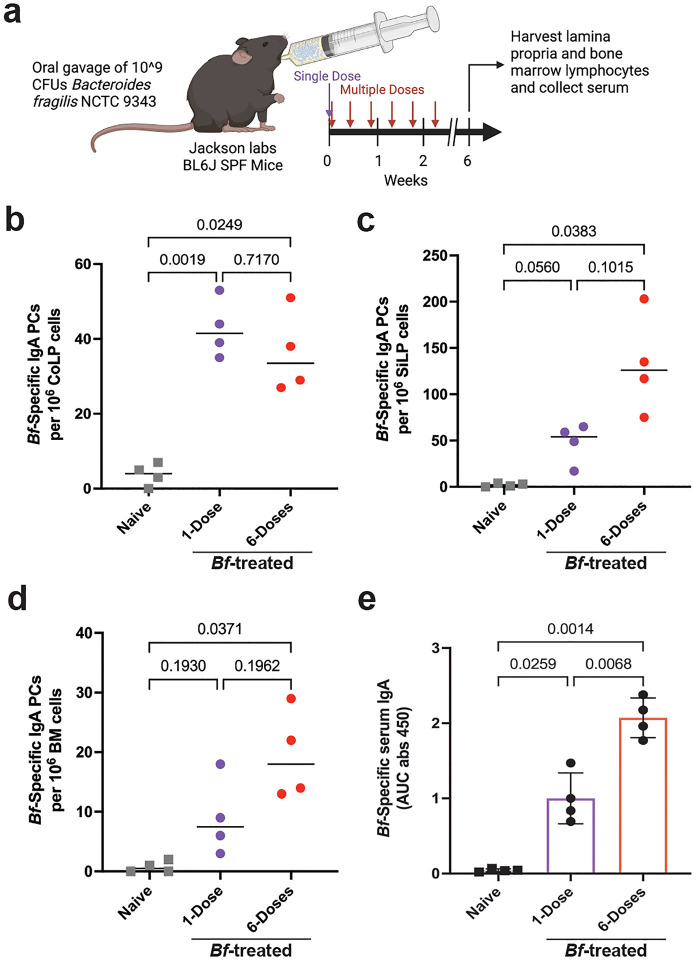
Oral gavage of *Bacteroides fragilis* induces systemic IgA Responses in B6-SPF mice. **a**, Experimental design, including oral gavage treatment strategy and plasma cell induction period. **b**,**c**, ELISpot assays of *B. fragilis*-specific mucosal IgA plasma cells isolated from colonic (**b**) and small intestinal (**c**) lamina propria 6-weeks after initial dose. **d**, ELISpot assay of *Bf*-specific systemic IgA plasma cells isolated from bone marrow 6-weeks after initial dose. **e**, Serum ELISA determined the level of *Bf*-specific IgA in *Bf*-treated mice. For the experiment shown, n=4 mice in each cohort. Plasma cell ELISpot and serum ELISA was performed using appropriate plates coated with heat-killed antigen from the tested bacteria. For serum ELISA, the area under the curve (AUC) was calculated based on the absorbance at 450 nm from each serially diluted sample. Statistical analysis was performed using one-way analysis of variance (ANOVA) with Turkey multiple-comparison test (**b**-**e**). Exact *P* values are shown.

*Bacteroides fragilis* (*Bf*) is known to induce organism-specific IgA responses in the gut mucosa using mouse model systems^13^. To test whether *Bf* is an efficient inducer of systemic IgA responses, we treated specific pathogen free C57Bl/6J mice sourced from Jackson Laboratories (B6-SPF) mice with a single- or multi-dose oral gavage regimen (Fig. 1a). After 6-weeks we assayed the induction of *Bf*-specific plasma cells by ELISpot assay and serum immunoglobulin by ELISA. In accordance with previous studies, we found that oral treatment with *Bf* led to the robust induction of *Bf*-specific colonic lamina propria (CoLP) IgA plasma cells (Fig. 1b^)13,17.^ Induction of *Bf*-specific CoLP IgA plasma cells occurred at similar levels regardless of single- or multi-dose exposure. This finding is in contrast with the small intestine lamina propria (SiLP) and bone marrow (BM), where only the multi-dose *Bf*-treatment resulted in significant increases in *Bf*-induced IgA plasma cell populations (Fig. 1c,d). *Bf* treatment was also found to induce a population of *Bf*-specific IgA plasma cells in the spleen (Extended Data Fig. 1a). Increased *Bf*-specific IgA plasma cells did not coincide with an increase in the frequency of total IgA plasma cells in the CoLP, SiLP, nor BM (Extended Data Fig. 1b–d). The frequency of commensal-specific IgA plasma cells in the bone marrow correlates with serum antibody titers^3^. We find that *Bf*-specific serum IgA increases following both single- and multi-dose *Bf* treatment with a larger increase in response to multi-dose compared to naïve controls (Fig. 1e). The presence of *Bf-*specific IgA in the serum is dependent upon exposure, however, we find serum IgG and IgM specific to *Bf* in the naïve controls (Extended Data Fig. 1e,f), likely due to cross reactivity with common gram-negative commensal bacterial antigens^18^.

**Extended Data Fig. 2.**
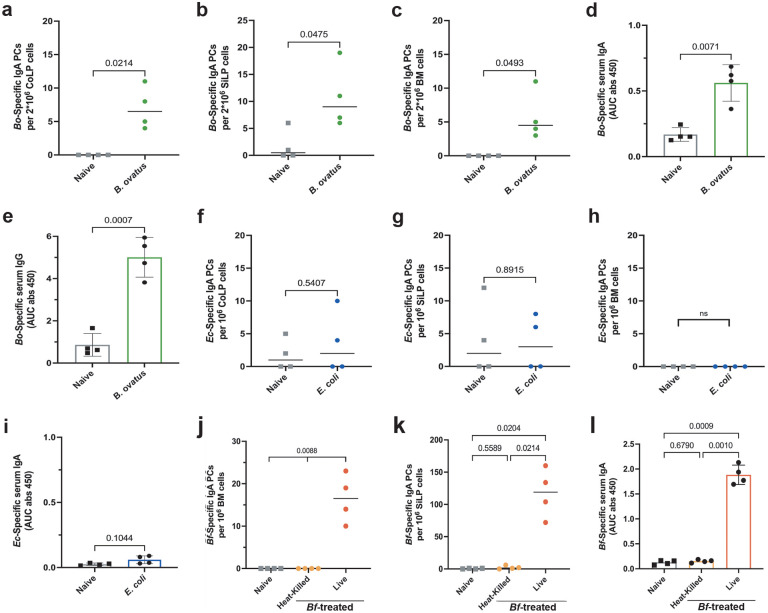
Treatment with live *B. fragilis* leads to robust IgA induction compared to other bacterial species. **a**,**b**,**c**, ELISPot assays of *B. ovatus*-specific IgA plasma cell induction from colon (**a**), small intestine (**b**), and bone marrow (**c**) of naïve and 6-dose treated mice. **d**,**e**, Serum ELISA determined the level of *Bo*-specific IgA (**d**) and IgG (**e**) from naive and 6-dose treated mice. **f**,**g**,**h**, ELISpot assays determined the frequency of *E. coli* K-12 specific IgA plasma cell populations in the colon following multi-dose treatment (**f**), small intestine (**g**), and bone marrow (**h**). **i**, Serum ELISA determined the level of *Ec*-specific IgA from naive and 6-dose treated mice. **j**,**k**, B6-SPF mice were given 6-doses of live or heat killed *B. fragilis* then bone marrow (**j**) and small intestine (**k**) IgA plasma cells were assayed with ELISpot to determine specificity to *Bf*. **l**, Serum ELISA was performed to detect induction of *Bf*-IgA in mice treated with heat-killed *Bf*. ELISpot and ELISA plates were pre-coated with heat-killed *B. ovatus*, *E. coli*, or *B. fragilis* and responses were assayed 6-weeks after initial treatment. For all experiments shown, n=4 mice in each cohort. For serum ELISA, the area under the curve (AUC) was calculated based on the absorbance at 450 nm from each serially diluted sample. Statistical analysis was performed using students t-test with Welch’s correction (**a-i**) or one-way analysis of variance (ANOVA) with Turkey multiple-comparison test (**j-l**). Exact *P* values are shown. Data are representative of three independent experiments.

A variety of commensal bacteria taxa have been demonstrated to be systemic IgA inducers in a polymicrobial model system^3^. To determine if induction of systemic IgA is a common response to bacteria when introduced by oral gavage, we treated mice with two additional human commensals species. We chose an additional commensal bacterium from the *Bacteroides* genus, *Bacteroides ovatus* (*Bo*), and an unrelated common laboratory strain of bacteria, *Escherichia coli* K12 (*Ec*). Like *Bf*, *Bo* has been reported to be an efficient inducer of colonic mucosal IgA responses^19^. Consistent with our findings for *Bf*, multi-dose treatment with *Bo* induced organism-specific CoLP, SiLP, and BM IgA plasma cells, however *Bo* induction was significantly less robust than *Bf* (Extended Data Fig. 2a–c). *Bo* treatment also led to the induction of organism-specific serum IgA, albeit at lower levels than *Bf*, and a significant increase in *Bo*-specific serum IgG (Extended Data Fig. 2d,e). Treatment with *Ec* did not significantly induce IgA responses in either the CoLP, SiLP, BM, or serum (Extended Data Fig. 2f–i). These data suggest that *Bf* is a particularly robust systemic IgA inducer and other commensals, including *Bo* and *Ec*, range from weak to no induction at all.

### *Bf* robustly colonizes murine GI tract

**Extended Data Fig. 3.**
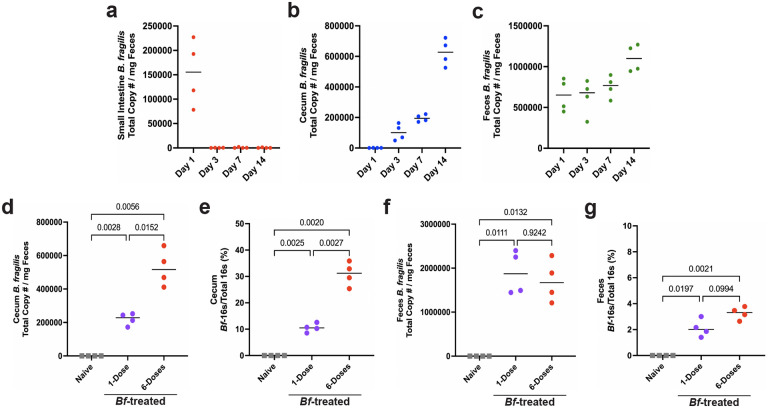
*Bacteroides fragilis* primarily colonizes the cecum and colon of Jax-SPF mice. **a**-**c**, qPCR analysis of *Bf* colonization following single-dose B6-SPF mice determined absolute copy number of *Bf* in the small intestine (**a**), cecum (**b**), and feces (**c**) 1-, 3-, 7-, and 14-days post-treatment. **d-e**, qPCR determination of *Bf* absolute copy number (**d**) and relative abundance (**e**) in cecum following single- and multi-dose treatment 6-weeks after initial dose. **f**-**g**, qPCR determination of *Bf* absolute copy number (**f**) and relative abundance (**g**) in feces following single- and multi-dose treatment 6-weeks after initial dose. Data representative of two independent experiments (n=4 mice per group).

**Fig. 2.**
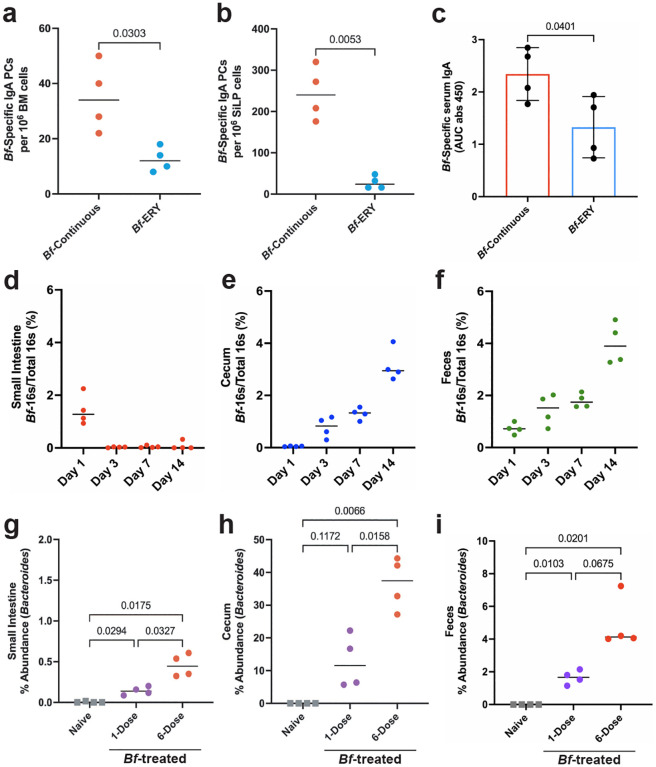
Systemic IgA induction by *B. fragilis* requires continuous colonization of B6-SPF mice. **a**,**b**, Necessity of continuous colonization for IgA generation was tested by treating mice with multi-dose *Bf* regimen followed by erythromycin (ERY) in drinking water for remainder of induction period. ELISpot assays of bone marrow (**a**) and small intestine (**b**) IgA plasma cells using plates pre-coated with heat-killed *Bf*. **c**, Serum ELISA determined induction of *Bf*-IgA in ERY-treated mice. **d-f**, qPCR analysis of *Bf* colonization following single-dose determined relative abundance of this bacterium in the small intestine (**d**), cecum (**e**), and feces (**f**) 1-, 3-, 7-, and 14-days post-treatment. **g**-**i**, qPCR determination of *Bf* relative abundance in small intestine (**g**), cecum (**h**) and feces (**i**) following single- and multi-dose treatment 6-weeks after initial dose. For all experiments shown, n=4 mice in each cohort. For serum ELISA, the area under the curve (AUC) was calculated based on the absorbance at 450 nm from each serially diluted sample. Statistical analysis was performed using students t-test with Welch’s correction (**a-c**) or one-way analysis of variance (ANOVA) with Turkey multiple-comparison test (**g**-i). Exact *P* values are shown. Data are representative of three independent experiments.

To determine if systemic IgA induction by *B. fragilis* requires colonization, we performed multi-dose treatment of B6-SPF mice with heat killed *Bf*. Treatment with heat killed *Bf* led to no induction of organism-specific mucosal or systemic IgA, suggesting live bacteria are required (Extended Data Fig. 2j–l). To assess if continuous colonization is required for systemic IgA induction, we provided mice with erythromycin in their drinking water after the multi-dose *Bf* treatment period. Antibiotic administration led to a significant reduction in *Bf*-specific IgA responses, suggesting continuous colonization by live bacteria is required for robust systemic IgA induction (Fig. 2a–c). We then asked about the dynamics of early colonization after a single oral dose. We performed qPCR on DNA isolated from small intestinal, cecal, and fecal contents to determine the abundance of *Bf* during the first two weeks of colonization (Fig. 2d–f, Extended Data Fig. 3a–c). In accordance with previous studies, we found that *Bf* primarily colonized the cecum and colon of mice, and single-dose treatment resulted in low levels of *Bf* in the SI compartment^20–21^.

**Extended Data Fig. 4.**
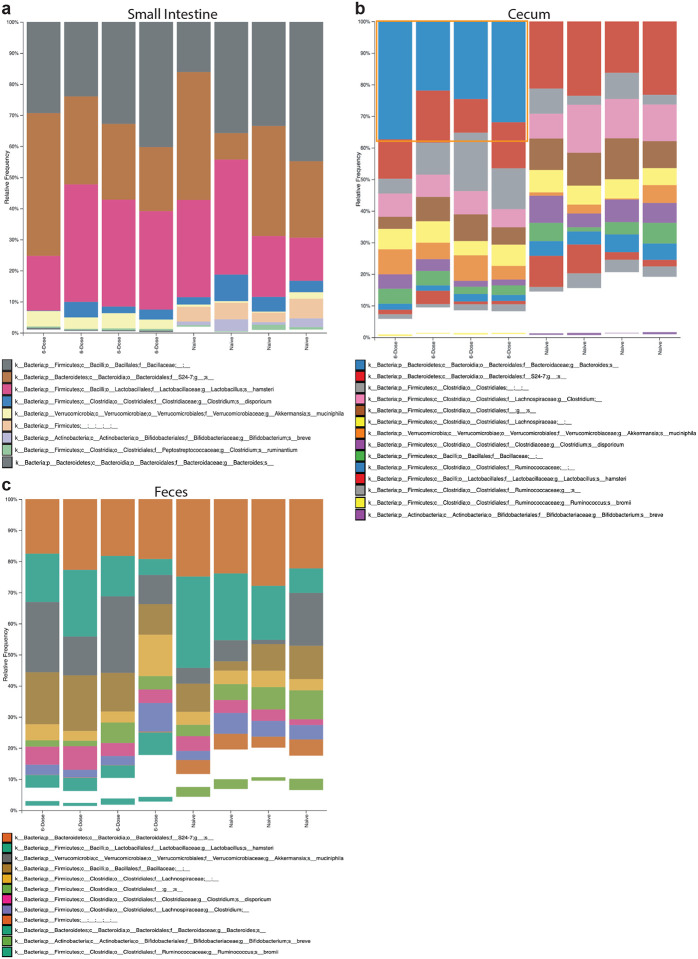
Minor changes in microbiota composition occur after *Bf* colonization. **a-c**, 16S rRNA taxonomic analysis of relative abundance of bacterial taxa in small intestine (**a**), cecum (**b**), and feces (**c**) after 6-weeks of multi-dose *Bf* colonization. Less abundant taxa (primarily *Clostridales*) that did not significantly change between cohorts were removed for simplicity. Increase of *Bf* in cecal populations denoted with orange box. NCBI nucleotide BLAST database confirmed the only reads assigned as *Bacteroides* species were *Bacteroides fragilis*. 6-Dose refers to *Bf* oral administration.

Given that continued colonization is required for robust IgA generation, we hypothesized that colonization dynamics could explain the differences in systemic IgA induction. We performed 16S rRNA sequencing on SI, cecal, and fecal contents from single and multi-dose treated mice and compared the relative abundance of *Bf* in these tissues (Fig. 2g–i). Importantly, we observed a statistically significant dose-dependent increase in *Bf* colonization in the SI and cecum, but not the feces. While *Bf-*SI abundance represented <1% of total bacteria, we saw a substantial increase in the cecum (>30%) that was not reflected in the feces. To confirm these findings using a targeted approach, we performed qPCR on cecal and fecal bacterial DNA using *Bf*-specific primers and observed similar abundance levels as seen in the 16S data (Extended Data Fig. 3d–g). Taxonomic classification of the overall microbial landscape revealed that beyond the large expansion in *Bf* cecal abundance, there were no major differences in the microbial composition of these mice apart from minor shifts in some of the less abundant taxa. (Extended Data Fig. 4a–c) Collectively, these data support a dose-dependent relationship between intestinal colonization by *Bf* and subsequent IgA responses.

### IgA induction by *Bf* does not require PSA or TLR2

**Extended Data Fig. 5.**
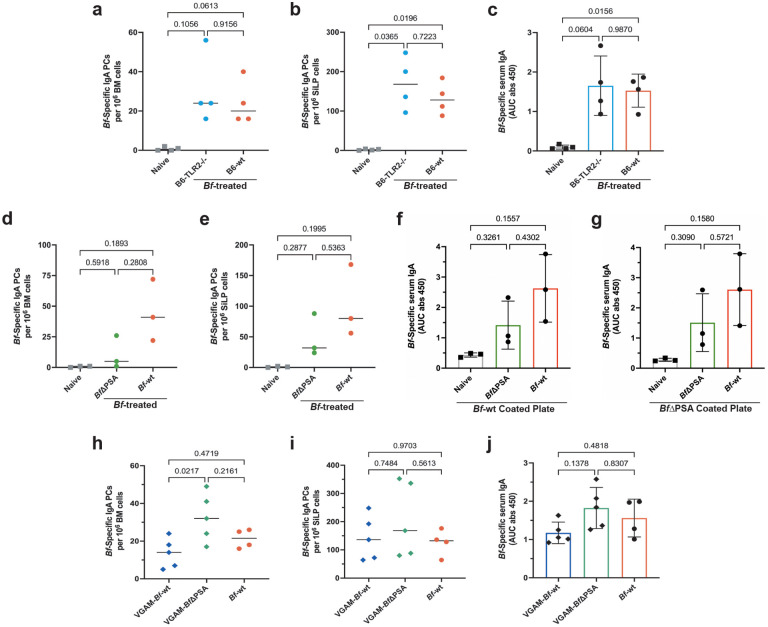
Systemic IgA induction by *Bf* does not require PSA or TLR2. **a**,**b**, ELISpot assays of *Bf*-specific IgA plasma cells isolated from BM (**a**) and SiLP (**b**) of *Bf*-treated B6-*Tlr2*^*−/−*^ and B6-wt mice. **c**, Serum ELISA was performed to determine the level of *Bf*-specific IgA from *Bf*-treated B6-*Tlr2*^*−/−*^ and B6-wt mice. **d**,**e**, ELISpot assays of *Bf*-specific IgA plasma cells isolated from BM (**d**) and SiLP (**e**) of *Bf*- and *Bf*ΔPSA-treated mice. **f**,**g**, Serum ELISA determined level of *Bf*-specific IgA in *Bf*- and *Bf*ΔPSA-treated mice. ELISA plates were coated in heat-killed *Bf*-wt (**f**) or *Bf*ΔPSA (**g**). **h**,**i**, ELISpot assays of *Bf*-specific IgA plasma cells isolated from BM (**h**) and SiLP (**i**) of *Bf*-wt- and *Bf*ΔPSA-treated mice that were pre-treated with VGAM (vancomycin, gentamicin, ampicillin, metronidazole). **j**, Serum ELISA determined level of *Bf*-specific IgA in *Bf*-wt- and *Bf*ΔPSA-treated mice that were pre-treated with VGAM. For all experiments shown, n=3–5 mice/group. For serum ELISA, area under the curve (AUC) was calculated based on the absorbance at 450 nm from each serially diluted sample. Statistical analysis was performed using one-way analysis of variance (ANOVA) with Turkey multiple-comparison test (**a**-**j**). Exact *P* values are shown. Data are representative of three independent experiments.

The bulk of the research regarding the immunomodulatory capacity of *B. fragilis* has focused on a component of its outer polysaccharide coat known as PSA (polysaccharide A^)10.^ Toll-like receptor 2 (TLR2) has been described as the primary host receptor for the PSA immunomodulation axis^22^. To determine if IgA induction by *Bf* uses this pathway, we analyzed B6-*Tlr2*^−/−^ mice given multi-dose oral *Bf*. There was no significant difference between wild-type and B6-*Tlr2*^−/−^ mice in terms of frequency of *Bf*-specific IgA PCs in the BM and SiLP, nor in the level of *Bf*-specific serum IgA (Extended Data Fig. 5a–c). Additionally, we sought to rule out the possibility of PSA interacting with the host through TLR2-independent mechanisms. Therefore, to determine if PSA plays a role in systemic IgA induction, we utilized a mutant strain lacking this component (*Bf*ΔPSA^)23.^ Mice were treated with a multi-dose regimen of wild-type *Bf* or *Bf*ΔPSA and assayed for *Bf*-specific IgA PC frequency in the BM and SiLP (Extended Data Fig. 5d,e). Additionally, we assayed for both *Bf*-wt- or *Bf*ΔPSA-specific serum IgA using plates coated in either heat-killed *Bf*-wt or *Bf*ΔPSA (Extended Data Fig. 5f,g). We observed a slight decrease in the IgA inductive capacity of *Bf*ΔPSA in BM and SiLP PC numbers, as well as serum IgA, but no difference between ELISA plates coated in *Bf*-wt or *Bf*ΔPSA .

Considering the *Bf*ΔPSA strain has been previously characterized as having colonization deficiencies, we repeated this experiment using mice pre-treated with an antibiotic cocktail of vancomycin, gentamycin, ampicillin, and metronidazole (VGAM) to boost the colonization capacity of *Bf*ΔPSA^22^. The number of *Bf-*-specific BM and SiLP IgA PCs in the VGAM-*Bf*ΔPSA treated mice was similar, if not higher, than the VGAM-*Bf*-wt controls (Extended Data Fig. 5h,i). Additionally, an increase in the Bf-specific serum IgA was observed in VGAM *Bf*ΔPSA treated mice (Extended Data Fig. 5j). Taken together, we conclude that the well-characterized *Bf*-PSA and TLR2 immunomodulatory pathway is not required for *Bf*-induced systemic IgA responses.

### *Bf*-IgA induction requires Peyer’s patch germinal centers

**Extended Data Fig. 6.**
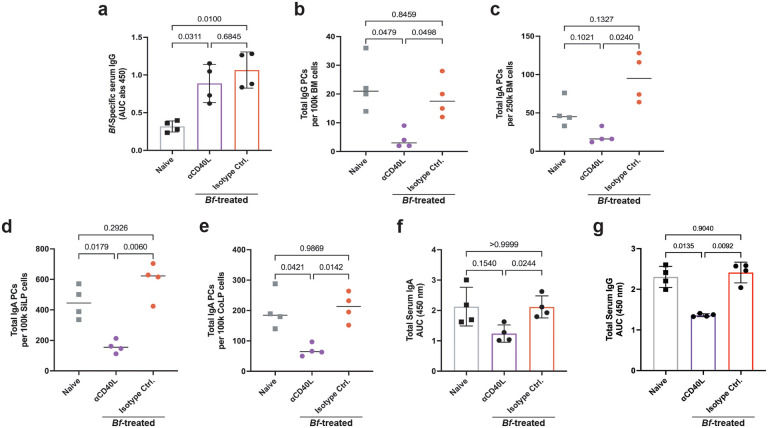
Anti-CD40L treatment reduces de novo plasma cell generation, but not *Bf-*IgG induction. **a**, *B. fragilis*-specific serum IgG ELISA of B6 mice treated with *Bf* multi-dose oral gavage and i.p. injections of MR1 (anti-CD40L). **b**,**c**, ELISpot analysis of total IgG (**b**) and IgA (**c**) plasma cell populations isolated from BM of MR1-treated mice. **d**,**f**, ELISpot analysis of total IgA plasma cell populations isolated from SiLP (**d**) and CoLP (**e**) tissues of MR1-treated mice. **f**,**g**, Serum ELISA of MR1-treated mice to assess total IgA (**f**) and IgG (**g**) levels. For experiment shown, n=4 mice/group. Plasma cell ELISpot and serum ELISA were performed using plates coated with heat-killed antigen from *Bf*. For serum ELISA, the area under the curve (AUC) was calculated based on the absorbance at 450 nm from each serially diluted sample. Statistical analysis was performed using one-way analysis of variance (ANOVA) with Turkey multiple-comparison test (**a**-**g**). Exact *P* values are shown. i.p., intraperitoneal.

**Fig. 3.**
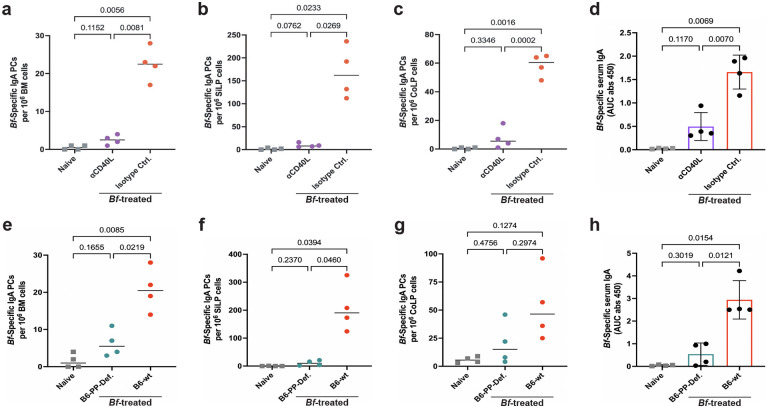
Generation of *Bf*-IgA requires Peyer’s patch germinal centers. **a**-**c**, *B. fragilis*-specific ELISpot analysis of IgA plasma cell populations isolated from BM (**a**), SiLP (**b**), and CoLP (**c**) tissues of B6 mice treated with *Bf* multi-dose oral gavage and i.p. injections of MR1 (anti-CD40L). **d**, *B. fragilis*-specific serum IgA ELISA of MR1-treated mice. **e**,**f**,**g**, Intestinal patch-deficient mice were treated with multi-dose *Bf* regimen and assayed for *Bf-*IgA plasma cells isolated from BM (**e**), SiLP (**f**), and CoLP (**g**). **h**, *Bf*-IgA ELISA using serum isolated from *Bf*-treated patch-deficient mice. For all experiments shown, n=4 mice/group. Plasma cell ELISpot and serum ELISA were performed using plates coated with heat-killed antigen from *Bf*. For serum ELISA, area under the curve (AUC) was calculated based on the absorbance at 450 nm from each serially diluted sample starting at 1:20. Statistical analysis was performed using one-way analysis of variance (ANOVA) with Turkey multiple-comparison test (**a**-**h**). Exact *P* values are shown. Data are representative of two independent experiments. i.p., intraperitoneal.

Both T cell-dependent and T cell-independent mechanisms for generation of gut-resident IgA plasma cells have been described^24^. However, systemic IgA plasma cells that home to the BM have been suggested to be T cell-dependent^3^. Therefore, we hypothesized that *Bf*-induced systemic IgA would be diminished by blocking CD40L-CD40 interactions between T and B cells in the germinal centers. We treated B6 mice with a multi-dose oral *Bf* regimen concurrent with anti-CD40L (MR1) or isotype control antibody. We found a near total ablation of *Bf*-specific IgA plasma cells in BM, SiLP, and CoLP in MR1-treated mice (Fig. 3a–c). Additionally, there was a significant decrease in *Bf*-specific serum IgA (Fig. 3d), but curiously, we observed no change in *Bf*-IgG following CD40L blockade (Extended Data Fig. 6a). These data suggest that CD40L blockade and subsequent germinal center disruption is detrimental to the development of *Bf-*specific systemic IgA, but not IgG. However, we observed significant decreases in the frequency of total IgG and IgA PCs in the BM, and total IgA PCs in the SiLP, and CoLP that correspond with decreased total serum IgA and IgG (Extended Data Fig. 6b–g). Together, these data suggest that systemic and mucosal *Bf*-specific IgA induction occurs primarily through a germinal center-dependent mechanism.

**Extended Data Fig. 7.**
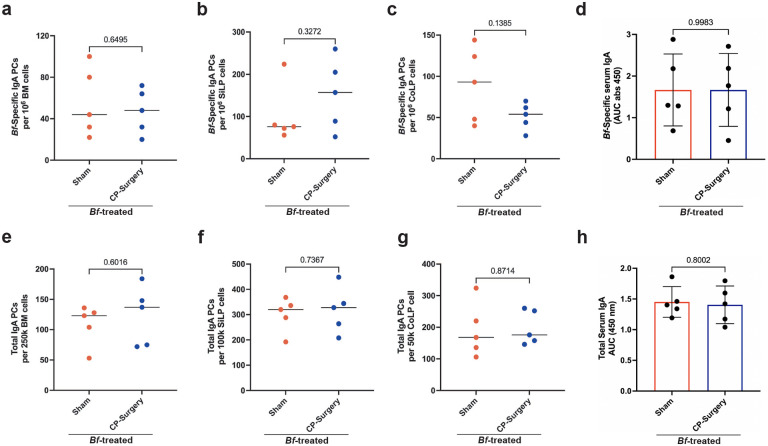
Cecal patch-deficient mice maintain capacity for *Bf*-specific IgA induction. **a**,**b**,**c**, ELISpot analysis of *Bf*-specific IgA plasma cell populations isolated from BM (**a**), SiLP (**b**), and CoLP (**c**) tissues of cecal patch-deficient B6 mice. **d**, Serum ELISA of cecal patch-deficient mice to assess generation of *Bf*-specific IgA. **e**,**f**,**g**, ELISpot analysis of total IgA plasma cell populations isolated from BM (**e**), SiLP (**f**), and CoLP (**g**) tissues of cecal patchdeficient B6 mice. **h**, Total serum IgA levels in cecal patch-deficient mice. For all experiments, n=5 mice/group. For serum ELISA, area under the curve (AUC) was calculated based on the absorbance at 450 nm from each serially diluted sample. Statistical analysis was performed using students t-test with Welch’s correction (**a-h).** Exact *P* values are shown.

We next sought to determine what germinal centers within the murine gut-associated lymphoid tissues (GALT) are responsible for *Bf*-induced IgA plasma cell expansion. Due to the high abundance of *Bf* colonization in the cecum compared to the small intestine and colon, we posited that the cecal patch may be a primary IgA plasma cell inductive site for systemic responses (Fig. 2). To test this, we performed partial appendectomy of B6-SPF mice to remove the cecal patch while leaving all other GALT intact. Following appendectomy, or sham surgery, mice were treated with oral multi-dose *Bf* and assayed for IgA responses. We found that a lack of cecal patches did not significantly affect the induction of systemic *Bf*-specific IgA plasma cells, however we saw slight increases in *Bf*-IgA PCs in the SI and slight decreases in the colon (Extended Data Fig. 7a–c), that are consistent with reported data in mice with their cecal patches removed^25^. Furthermore, we saw no difference in *Bf*-specific serum IgA between experimental and sham groups nor did we observe any overall changes in total IgA generation in the intestines, bone marrow, or serum (Extended Data 6d-h).

**Extended Data Fig. 8.**
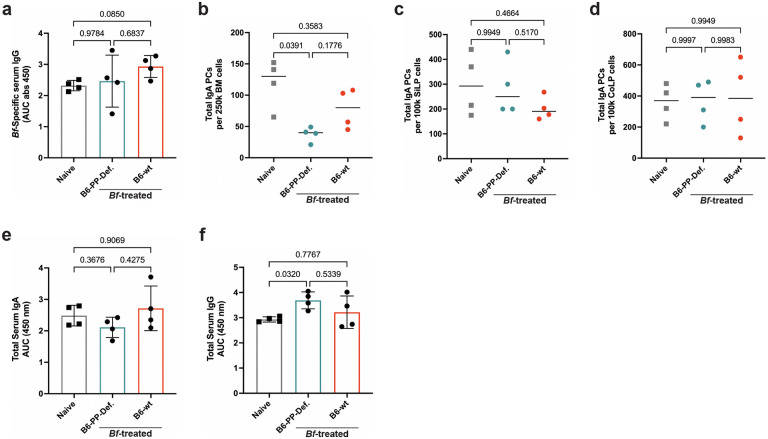
Peyer’s patch-deficient mice retain competent plasma cell induction mechanisms. **a**, *B. fragilis*-specific serum IgG ELISA of patch-deficient B6 mice treated with *Bf* multi-dose oral gavage. **b**,**c**,**d**, ELISpot analysis of total IgA plasma cell populations isolated from BM (**b**), SiLP (**c**) and CoLP (**d**) tissues of PP-deficient B6 mice. **e**,**f**, Serum ELISA of PP-deficient mice to assess total levels of IgA (**e**) and IgG (**f**). For all experiments shown, n=4 mice/group. For serum ELISA, area under the curve (AUC) was calculated based on the absorbance at 450 nm from each serially diluted sample, Statistical analysis was performed using one-way analysis of variance (ANOVA) with Turkey multiple-comparison test (**a**-**f**). Exact *P* values are shown.

In light of these results, we turned our attention to small intestinal Peyer’s patches (PP), which were previously described to be required for IgA PC migration to mammary tissues [4]. To test the necessity of PP in *Bf* systemic IgA induction, we used an established method of intestinal-patch depletion of B6 mice via treatment with an anti-IL-7Rα antibody during fetal development^26^. The anti-IL-7Rα antibody resulted in pups that lacked Peyer’s patches for the lifetime of the animal, while maintaining proficient isolated lymphoid follicle generation^27^. After 6-weeks, the resulting PP-deficient litters were subjected to multi-dose oral treatment with *Bf* along with age/sex-matched control mice. We saw a substantial decrease in the frequency of *Bf*-specific IgA PCs in the BM and SiLP, but not the CoLP in mice lacking Peyer’s patches (Fig. 3e g). The reduced frequency of *Bf*-specific IgA PCs coincided with a decrease in *Bf*-specific serum IgA in PP-deficient mice, yet similar levels of *Bf*-specific IgG were observed in both groups (Fig. 3h, Extended Data Fig. 8a). Furthermore, the lack of Peyer’s patches had little effect on the levels of total PC populations in the SiLP and CoLP, however we observed a decrease in total BM IgA PCs in Peyer’s patch-deficient mice compared to naïve controls (Extended Data Fig. 8b–d). Similarly, we observed no significant change in total serum IgA in patch-deficient mice and total serum IgG was higher compared to the naïve controls, therefore, these data suggest these mice supported relatively normal PC induction mechanisms (Extended Data Fig. 8e–f). Collectively, these findings suggest that induction of high-levels of *Bf*-specific systemic IgA is dependent on Peyer’s patch germinal centers.

### *Bf* colonization elicits minor phenotypic changes in the gut

**Extended Data Fig. 9.**
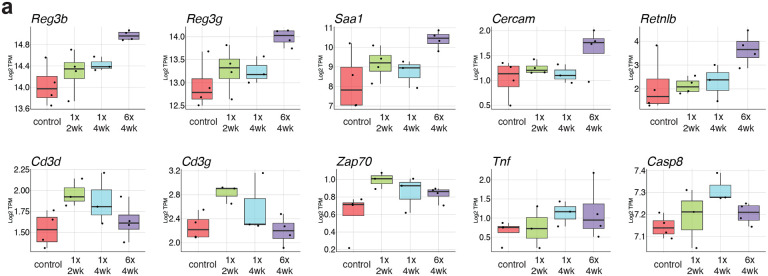
Multi-dose *B. fragilis* treatment induces antimicrobial gene expression in the small intestine. **a.** Magnitude of gene expression as log2 TPM is shown for selected genes and displayed as the mean (bar), 75% confidence interval (box), 95% confidence interval (whisker), and individual sample data points (jitter) for each small intestine treatment group.

**Fig. 4.**
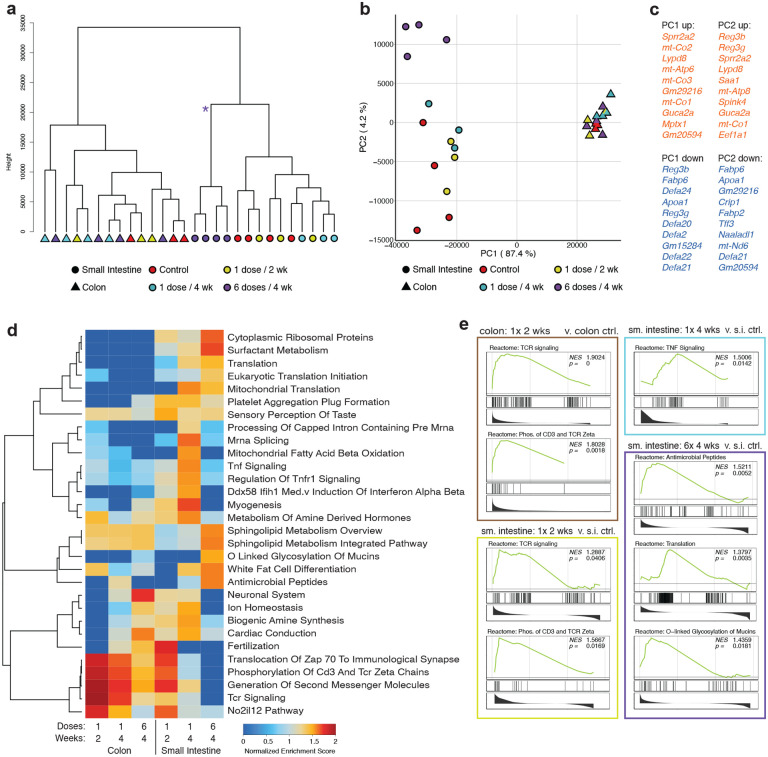
*B. fragilis* colonization elicits minor transcriptional changes in the gut. Transcriptomic analysis was performed on colon and small intestine tissue of animals treated with a single or multiple doses of *Bf* harvested 2 or 4 weeks post initial treatment. **a.** Hierarchical clustering of all samples is shown with shapes indicating tissue of origin and color indicating treatment groups. Asterisk (*) indicates branch point of small intestine multi-dose group. **b.** Principal component analysis is displayed for PC1 and PC2. **c.** Primary loading (top ten up and down) genes are shown for PC1 and PC2. **d., e.** GSEA was performed for all curated pathway genesets comparing each sample group to relevant tissue control. **d.** All significantly (p<.05) enriched pathways are shown as a heatmap of normalized enrichment scores. **e.** Selected enrichment plots are shown for enriched pathways and are grouped by comparison of enrichment.

To test if treatment with *Bf* leads to phenotypic changes in the gut that could provide insight into the mechanisms of systemic IgA induction, we performed a series of experiments to characterize the gut phenotype after single- or multi-dose treatment. We performed RNA-seq on tissue from the small intestinal ileum or proximal colon at either 2 or 4 weeks following the initial oral gavage with 1 or 6 doses of *Bf*. The largest differences were observed between the two tissues, regardless of dosage, with small intestine and colon samples on opposing trees following hierarchal clustering (Fig. 4a). Principal component analysis showed a tightly grouped cluster of colon samples with some treatment specific variation amongst the small intestine groups (Fig. 4b). The genes driving the bulk of the difference along principal component 1 included a large metabolic signature and a subset of canonical colon genes, such as antimicrobial peptides, many of these canonical colon genes are upregulated in the small intestine following *Bf* treatment (Fig. 4c). Gene Set Enrichment Analysis (GSEA) showed a link between *Bf* dosage and T cell signaling, that waned following multi-dose treatment in both the small intestine and colon (Fig. 4d–e, Extended Data Fig. 9a). Multi-dose treatment increased the activation of cells in the small intestine leading to increased expression of genes related to translation, antimicrobial peptides, mucin glycosylation, and surfactant production (Fig. 4d, Extended Data Fig. 9a). These data indicate that there is a modest effect of multi-dose *Bf* treatment on the homeostasis of the gut, with the largest change occurring in the ileum of the small intestine. To confirm there was no overall change in the immune cell landscape within the small intestine and colon lamina propria, we performed multiparameter flow cytometry. We found no changes in the frequencies of neutrophils, dendritic cells, macrophages, B cells, plasma cells, CD4+ T cells, CD8+ T cells, or Th17 T cells compared to controls (data not shown). These data suggest that *Bf* is acting as a commensal bacterium, as shown by the minor phenotypic changes observed induced by colonization.

### *Bf-*IgA protects against abscess formation

**Fig. 5.**
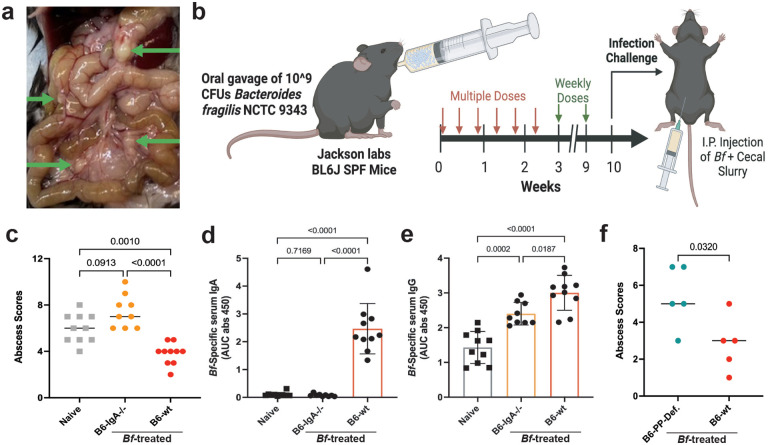
*B*. *fragilis* systemic IgA protects against peritoneal abscess formation. **a**, Intraperitoneal injection of live *B. fragilis* combined with inactivated cecal slurry leads to the formation of enumerable peritoneal abscesses (6-days after injection). **b,** Schematic of 10-week *Bf*-IgA induction procedure followed by infection challenge. **c**, Abscess scores for B6-wt and B6-*IgA*^−/−^ mice pre-treated with *Bf* for 10 weeks then challenged with *Bf*/cecal slurry. **d**-**e**, Serum ELISA was performed to determine the level of *Bf*-specific IgA (**d**) and IgG (**e**) in naïve and *Bf* pre-treated B6-*IgA*^−/−^ and B6-wt mice prior to abscess challenge. Naïve n=10, B6-*IgA*^−/−^ n=9, B6-wt n=10. **f**, Abscess scores for B6-PP-deficient and B6-wt mice pre-treated with *Bf*. B6 PP-deficient n=5, B6-wt n=5. Statistical analysis was performed using one-way analysis of variance (ANOVA) with Turkey multiple-comparison test (**c**-**e**) or students t-test with Welch’s correction (**f**). Exact *P* values are shown. Data are representative of two independent experiments.

*Bacteroides fragilis* NTC9343 is a nontoxigenic strain that has been investigated for numerous beneficial and possibly symbiotic effects on its host^14^. Despite this symbiosis, *Bf* is commonly implicated in several pathologies, most notably peritoneal abscess formation following bowel perforation^7^. We adapted a rat peritoneal abscess model for use in our murine system to test the potential functional role of *Bf*-specific systemic IgA responses^28^. Using live *Bf* in combination with inactivated cecal slurry, we can mimic the conditions of polymicrobial peritoneal abscess formation, whilst eliminating the confounding effects of multiple species of live bacteria. Intraperitoneal injection of inactivated cecal slurry mixed with axenic culture of *Bf* leads to the formation of numerous peritoneal abscesses that can be grossly enumerated upon necropsy (Fig. 5a). To determine if *Bf*-specific IgA plays a protective role from peritoneal abscess formation, we treated wildtype B6 and B6-*IgA*^−/−^ mice with our multi-dose regimen of *Bf* by oral gavage, along with subsequent weekly doses to establish maximal *Bf*-specific systemic IgA induction. After 10 weeks of oral *Bf* pre-treatment, naïve (non-treated), B6-wt, and B6-*IgA*^−/−^ mice were given a single intraperitoneal (i.p.) injection of *Bf* and inactivated cecal slurry to induce peritoneal abscess formation (Fig. 5d). B6-*IgA*^−/−^ and naïve B6 controls displayed significantly higher frequencies of peritoneal abscess compared to *Bf*-pretreated B6-wt mice (Fig. 5c). The reduction in abscess formation observed in *Bf*-pretreated B6-wt mice correlates with high levels of *Bf*-specific serum IgA (Fig. 5d). Despite significant titers of *Bf*-specific IgG, B6-*IgA*^−/−^ mice were equally susceptible to abscess formation as naive mice (Fig. 5e). Additionally, when PP-deficient mice were pretreated with *Bf* then subjected to peritoneal abscess induction, we observed a significantly greater number of abscesses compared to wild-type *Bf*-pretreated controls (Fig. 5f). In summation, these data suggest that *Bf*-specific systemic IgA induced by intestinal *Bf* colonization protects against peritoneal abscess formation.

## Discussion

Our work shows that the human commensal *Bacteroides fragilis* can induce a robust serum IgA response resulting in protection from excessive peritoneal abscess formation. Furthermore, robust systemic IgA responses to *Bf* require repeated exposure provided by multi-dose treatment that must occur in the presence of Peyer’s patches and germinal centers. Although a single oral dose was sufficient to elicit low-level responses, we found a multi-dose strategy significantly enhanced SI and BM IgA PC generation and serum IgA. Clinical applications that employ fecal microbiota transplantation commonly utilize a multi-dose modality to overcome the barriers of autochthonous microbes^29^. By utilizing mice with a conventional microbiome, we have modeled *Bf* treatment that mirrors multi-dose probiotic administration allowing us to better recapitulate the dynamics of potential therapeutic intervention. Similar to gastrointestinal IgA responses to microbiota, we found that systemic IgA increased in an additive manner based on dosage, which differs from the prime-boost nature of memory IgG responses^30–31^. Dose-dependent enhancement of IgA induction paralleled increased abundance of *Bf* in the cecum. The dissimilarity of *Bf* abundance between the cecum and feces suggests that microbiome analysis of fecal contents is not always an accurate reflection of microbial composition in other regions of the gastrointestinal tract. Future studies exploring the effects of microbiome modulation may benefit from the application of multiple oral treatments and analysis of different regions of the intestinal tract.

Bacterial capsular polysaccharides (CPs), such as PSA, have the capacity to ameliorate or exacerbate pathology based on the physiological context^7^. Previous studies have defined the *Bf*-PSA-TLR2 axis as a central immunomodulatory mechanism for regulating T cell imbalances and generating anti-inflammatory IL-10^22,32^. PSA can be packaged into outer membrane vesicles (OMVs) and translocated to mesenteric lymph nodes (MLNs) by dendritic cells^33–34^. Although our work demonstrated TLR2 and PSA are not required for systemic IgA induction, the role of dendritic cells, OMVs, and MLN-translocation is still unclear in this process. While systemic induction of IL-10-producing immune cells by CPs has been shown to alleviate numerous diseases states, local induction of IL-10 and the subsequent dampening of innate immune cell recruitment can prevent clearance of abscess formation^35–36^. Bacterial CPs further assist in innate immune evasion by preventing formation of complement attack complexes^37^. The contradictory roles of *Bf* as both pathogen and symbiont raise the question as to why the host species maintains homeostasis with *Bf* during initial and prolonged colonization. Considering these facts, we propose systemic IgA serves as a non-inflammatory secondary containment measure to prevent dissemination in cases where the epithelial lining of the gut is jeopardized. In this way, the host can conserve the symbiotic effects of *B. fragilis* and mitigate the risks associated with close-epithelial colonization.

Previous reports have demonstrated that passive transfer of immunoglobulins from rats immunized with *Bf*-CP did not protect against abscess formation in naive animals^38^. We believe this is due to the inflammatory nature of IgG-Fcγ receptor interactions and the pathophysiology of abscess formation^39^. In humans, serum IgA interacting with the Fcα receptor (CD89), has been shown to exhibit non-inflammatory properties while maintaining the ability to opsonize and neutralize pathogens^40^. Our work in mouse model systems supports the hypothesis that systemic IgA functions as a non-inflammatory mediator by preventing mortality from sepsis^3^. Microbes that colonize their host near the intestinal epithelial layer often show high immunogenicity and a propensity to elicit T cell-dependent mucosal IgA responses^13,15–16^. We believe that organism-specific systemic IgA responses are a result of direct exposure of gut-associated lymphoid tissues to mucosal penetrant microbes. Moving forward, identification of the specific antigenic targets of systemic IgA may elucidate the underlying immune mechanisms. We suggest that defining these pathways will reveal a deeper understanding of host-microbe interactions and enhance therapeutic approaches, such as mucosal vaccine design.

## Methods

### Mice –

All mice used in this study were maintained at the Upstate Medical University vivarium under specific pathogen free conditions. B6 mice termed ‘B6-SPF’ originated from Jackson Laboratories and housed in isolation from other mice to prevent transmission of additional microbes. C57Bl/6J and B6.129-*Tlr2*^*tm1Kir*^/J (B6-*Tlr2*^−/−^) mice were purchased from Jackson Laboratories. B6-*IgA*^−/−^ mice were kindly provided by the University of Pennsylvania with permission from Dr. Margaret Connor (Baylor University) and were bred and maintained at Upstate Medical University. To control for potential microbiome differences, B6-*IgA*^−/−^ mice were treated with VGAM; vancomycin (500μg/mL), gentamicin (500μg/mL), ampicillin (1mg/ml), metronidazole (1mg/mL), and sucrose (2mg/ml) in drinking water *ad libitum* for 10 days, followed by co-housing with aged matched C57Bl/6J mice from Jackson Laboratories. For experiments involving erythromycin, a concentration of 25μg/mL was provided in drinking water *ad libitum*. All mice for this study were housed in autoclaved cages with autoclaved bedding/water and irradiated food. Most experiments utilized female mice to facilitate cohousing; except for anti-IL-7Rα experiments, which used both male and female mice. Mice were 6–8 weeks old at experiment onset and aged 8–20 weeks at euthanasia. All experiments were performed in accordance with the Office of Regulatory Affairs Institutional Animal Care and Use Committee.

### Generation of Intestinal Patch-Deficient Mice –

Male and female C57BL/6J (B6) mice were purchased from Jackson Laboratories and mated overnight (~8-hours), then females that exhibited significant weight gain after 12-days were deemed pregnant. At 14.5 days post-coitus, pregnant dams were given an i.p. injection of 1-mg of anti-IL-7Rα, as previously described^26^. The resulting offspring were weaned after 3 weeks and subsequently treated with *B. fragilis* by oral gavage starting at 6–8 weeks of age. Impairment of Peyer’s patch development was confirmed by post-mortem dissection of intestinal tissues.

### Cecal Patch Partial Appendectomy –

Appendectomy surgeries were performed as previously described by Li et al^41^. In summary, C57BL/6J (B6) mice were anesthetized using 3% isoflurane and the incision site was prepared by shaving the area and sterilizing with iodine scrub. A 2cm incision was made along the superior midline of the abdominal cavity. The distal portion of the cecum was gently pulled through the incision and the mesenteric vessels were ligated with 6–0 silk suture (Demetech). The region of cecal resection was demarcated with an open loop suture and ~1cm of the distal cecum was excised. Exposed cecal contents were removed with iodine swabs and the area was irrigated with sterile PBS. The cecal stump was then closed with a rolling, continuous suture (6–0). This procedure ensured the cecal patch was completely removed while minimizing contamination. The remaining cecum was gently reinserted into the abdominal cavity and the peritoneum was closed using 6–0 suture. The original incision was then closed with 4–0 silk suture (Coviden Sofsilk) and sterilized with iodine. Sham control groups underwent a similar procedure, except mesenteric vessels were not ligated and a single incision was made in the cecum, contralateral to the cecal patch. Mice were kept in pathogen free conditions and monitored daily for 14 days prior to removal of external sutures.

### In-vivo anti-CD40L Treatment –

C57BL/6J mice were injected i.p. with anti CD40L (MR1, Bio-X-cell) (100μg per mouse per injection) every three days for the entirety of the experimental period (6-weeks)^42.^ Vehicle control C57BL/6J mice were given i.p. injections of Armenian hamster polyclonal IgG (Bio-X-Cell) (100μg per mouse per injection).

### Bacterial strains –

*Bacteroides fragilis* (ATCC 25285, NTC9343) was cultured on brain heart infusion media (Anaerobe Systems PRAS BHI AS-6426) and isolates were incubated for 18–30 hours at 37° C under microaerophilic conditions (Pack-Micro, Mitsubishi Gas Chemical America Inc R681005). The *Bacteroides fragilis* PSA knockout strain (*Bf*ΔPSA) was generously provided by Dr. Laurie Comstock (University of Chicago) and was cultured following the same methods described for the *Bf* wild-type strain. *Bacteroides ovatus* (ATCC 8483) cultured on brain heart infusion media (Anaerobe Systems PRAS BHI AS-6426) and isolates were incubated for 24–48 hours at 37° C under microaerophilic conditions (Pack-Micro, Mitsubishi Gas Chemical America Inc R681005). *Escherichia coli* K-12 (ATC 25401) was cultured on Lennox Broth [LB] (Molecular Genetics CAS 8013-01-2) and isolates were incubated for 24 hours at 37° C under aerobic conditions. Pure cultures were harvested and resuspended in liquid BHI (or LB) media and measurement of bacterial density by weight was based on colony formation unit (CFU) counts. For all bacterial gavages, mice were given 10^9 CFUs (5-mg for *B. fragilis*), resuspended in 100-μL of sterile PBS, and administered via a 24-guage oral gavage needle (Pet Surgical MDAFN2438S). For heat-killed experiments, *Bf* at a concentration of 10^9 CFUs/100μL sterile PBS was heated to 65°C for 1-hour and stored in 1-mL aliquots at −20C until needed.

### Tissue preparation –

Isolation of splenocytes was carried out via mechanical disruption using frosted glass slides followed by filtration through 65 μm Nitex nylon mesh. Bone marrow cells were isolated by flushing femur and tibia bones with a 23-G needle and syringe filled with FACS buffer (PBS, 0.5% BSA, 1mM EDTA) followed by filtration through 65 μm Nitex nylon mesh. SiLP and CoLP preparations were performed using a modified protocol from Hall et al.^43^. Intestines were harvested and Peyer’s patches (for Si), fat, and intestinal contents were removed. The tissue was cut into ~2cm pieces then incubated at 37°C on a shaker for 22 minutes in RPMI 1640 supplemented with 20mM HEPES, 5mM EDTA, 2mM DTT, Penicillin/Streptomycin, and 5% FBS. Samples were then washed in RPMI with Pen/Strep, 2mM EDTA, and 20 mM HEPES then minced and digested in RPMI with Pen/Strep, 20 mM HEPES, 0.1 mg/mL Liberase TL (Roche), and 0.05% DNase I (Sigma D5025) for 30 minutes at 37°C with shaking (225 RPM). The digestion reaction was then quenched with cold RPMI with 10% FBS, 0.05% DNAse I, Pen/Strep, 20 mM HEPES and the intestines were filtered through 70 μm cell strainers (Corning 352350). Lastly, isolated intestinal lymphocytes were filtered using 65 μm Nitex nylon mesh prior to plating for ELISpot or staining for flow cytometry^44–45^.

### ELISA/ELISpot assays –

ELISA and ELISpot assays were performed following standard protocols. Briefly, for ELISA, plates (ThermoFisher 442404) were coated with either heat-killed (65°C for 1-hr) bacterial antigen (500ng/mL) or anti-mouse Ig[H+L] (1μg/mL) (Southern Biotech 1010–01) in a sodium carbonate/bicarbonate solution pH 9.6. For organism-specific Ig responses, serum was plated at an initial dilution of 1:20. For total Ig quantification, serum was plated at initial dilution of 1:2000 for IgA and 1:10,000 for IgG. Plated serum was serially diluted and detected using biotinylated anti-mouse IgA/G/M antibody (Southern Biotech 1040–8/1030–8/1020–8). Biotinylated antibodies were revealed with streptavidin-HRP (Biolegend 405210) and BD OptEIA TMB substrate (BD 555214). ELISA plates were read at an absorbance of 450nm using a Vmax Kinetic Microplate Reader (Molecular Devices). ELISpot plates (EMD Millipore MSIPS4W10) were coated in the same manner as ELISA plates. Plated cells were serially diluted then incubated overnight (18-hrs) at 37°C/ 5% CO_2_. Detection was performed with biotin conjugated anti-IgA/G antibodies (Southern Biotech) then ExtrAvidin-alkaline phosphatase (Sigma E2636) and developed with BCIP/NBT (Sigma B1911). ELISpot plates were imaged, and spots were counted using a CTL Immunospot Analyzer (Cellular Technologies Limited).

### Intestinal Content Extraction and Bacterial DNA Isolation –

Extraction of small intestinal contents was carried out by first excising the small intestine 1cm proximal to the ileocecal junction to prevent contamination from the cecum. Contents were then expunged into a sterile centrifuge tube in an antegrade direction starting at the distal jejunum. Cecal contents were collected through resection of the distal cecal tip, followed by extraction by applying gentle pressure on the mid-portion of the cecum proper. Lastly, feces from mice were collected using standard methods directly prior to euthanasia. Intestinal contents/feces were weighed, then bacterial DNA was extracted using the QIAamp PowerFecal Pro DNA Kit (Qiagen 51804). The concentration of isolated bacterial DNA was quantified using a Qubit 4 Fluorometer (Invitrogen) and samples were stored at −80°C.

### Quantitative PCR –

Verification and enumeration of *Bacteroides fragilis* colonization via qPCR was achieved through amplification of the 16S rRNA gene regions of the *B. fragilis* genome (primer pairs Bf.qPCR.f; TCRGGAAGAAAGCTTGCT and Bf.qPCR.r; CATCCTTTACCGGAATCCT)^46^. Total bacterial load was determined using primers targeting universally conserved regions of bacterial 16S rRNA genes (16S.341.f; CCTACGGGAGGCAGC and 16S.806.r; GGACTACHVGGGTWTCTAAT)^47^. Reaction mixtures contained 25μL *Power*SYBR Green PCR Master Mix (Applied Biosystems), 200nM of both forward and reverse primers, and 10ng of sample DNA (50μL total Volume). Amplification was performed using an Applied Biosystems StepOnePlus Real-Time PCR System and a run method consisting of initial denaturation for 10 minutes at 95°C followed by 40 two-step cycles at 95°C for 15s and 60°C for 60s. In each run, negative template controls were included, melt curves were performed to confirm amplification of the correct product, and pure isolated DNA from *B. fragilis* culture was used to generate standard curves. Copy number per mg of feces/intestinal contents was calculated using measured Ct values for each sample (in duplicate). Relative abundance of *B. fragilis* was calculated by comparing *Bf* copy number per mg of feces to total bacterial copy number per mg feces.

### 16S sequencing –

Bacterial genomic DNA from intestinal contents and feces were isolated following the previously described methods followed by submission to the University of Buffalo Genomics and Bioinformatics Core. Degenerate V3V4 oligonucleotides and KAPA Biosystems HiFi PCR were utilized to amplify the V3V4 regions of bacterial 16s genes to produce 400 bp amplicons. The 16S amplicons were barcoded with Ilumina Nextera XT indexes for multiplexing through an additional round of HiFi PCR. Cleanup using Ampure beads was performed then final libraries were quantified using Qubit Fluorescence and Agilent Fragment Analyzer Visualization. Normalized libraries were pooled and sequenced using Illumina MiSeq platform. Amplicons were sequenced with paired end read lengths of 300 bp and were monitored for quality in real-time and upon completion of sequencing.

### Host-Intestinal RNA sequencing –

Experimental cohorts consisted of a 1-dose group treated 2-weeks prior to euthanasia, a 1-dose group treated 4-weeks prior, and a 6-dose group with a 2-week treatment period followed by a 2-week colonization period (4-weeks total). Small intestinal samples consisted of 1cm portions of the ileum and 1cm colon sections were taken starting 2cm distal to the cecum. Upon removal, intestinal samples were flash-frozen using dry ice and ethanol then stored at −80°C prior to processing. Isolation of RNA was accomplished following standard TRIZOL RNA isolation protocol using a glass Teflon homogenizer^48^. Isolated RNA was sent to Admera Health LLC for downstream processing. RNA-seq libraries were constructed using NEB Next Ultra II kit with Poly A section. Sequencing was run on an Illumina HiSeq at 2×150bp with 40 millions reads per sample (20M in each direction).

### Peritoneal Abscess Infection Model –

The *B. fragilis*-induced peritoneal abscess model was developed based on a previous model developed by Tzianabos, 1995^28^. Abiotic cecal slurry was created by removing cecal contents from euthanized mice followed by resuspension in PBS at 1mg cecal contents/ 10μl PBS. Resuspended slurry was then filtered through a 70μm Corning cell strainer and autoclaved. Autoclaved slurry was then filtered through 65μm nitex mesh and combined with axenic resuspension of *B. fragilis* in sterile PBS (50μL *Bf* [5*10^8 CFUs]/ 100μL cecal slurry per dose). Experimental mice for this procedure were given oral gavages of *B. fragilis* every 3 days for 15 days (6-doses) followed by weekly doses for an additional 7-weeks (13 total doses). One-week prior to infection challenge, mice were minimally bled (<50μL) to obtain serum for ELISA assays. After 10-weeks from the initial dose, mice were given 150μL i.p. injections of *Bf*/cecal slurry and closely monitored for 6-days. After this period, mice were euthanized, and peritoneal abscesses were enumerated by a blinded evaluator.

## Quantification and Statistical Analysis

### Statistical analysis –

The data presented were analyzed with Prism v10 software (GraphPad) for statistical analysis. Data are presented as mean ± SD and exact *P* values are shown. *P* values were determined using a two-tailed unpaired student’s t-test with Welch’s correction or Brown Forsythe and Welch one-way ANOVA with Turkey’s posttest where appropriate.

### 16S Sequencing analysis –

Sequencing data was initially checked for quality using the FASTQC software package^49^. Sequences were then trimmed and cropped to remove Illumina adapters using Trimmomatic V0.32 software^50^. Taxonomic classification and diversity analysis was accomplished using the QIIME 2–2020.11 software package performed with the command line interface and qiime plugins run in R^51^. Demultiplexed sequences were imported and denoised using DADA2 plugin. Paired-end amplicon sequence variants were merged and chimeric reads were removed from further analysis. A minimum of 10,000 merged sequences were analyzed for each individual sample. To identify unique amplicon sequence variants, we used a naïve Bayes machine-learning classifier trained on the Greengenes 13_8 reference database^52^. Taxonomic visualization was accomplished using the q2-feature-table plugin. Alpha and beta diversity analyses were performed using the q2-diversity plugin and the qiime diversity core-metrics-phylogenetic method.

## RNA Sequencing analysis

### Pseudoalignment and Gene expression –

Transcript abundance was computed by pseudoalignment with Kallisto^53^. Transcript per million (TPM) values were then normalized and fitted to a linear model by empirical Bayes method with the Voom and Limma R packages^54–55^ and differential gene expression was defined as a Benjemini and Hochberg corrected p-value of < 0.05 and fold change > 1 unless otherwise noted.

### Gene Set Enrichment Analysis –

GSEA was performed using the GSEA 4.3.2 tool (Broad Institute/ UCSD). Curated pathways (MSigDB: M2CP) with significant enrichment (p<.05) in any treated vs. control comparison using 1000 geneset permutations were chosen to display as a heatmap of normalized enrichment score. Data display was performed using the R statistical environment.

## Extended Data
